# Blinatumomab, a Bi-Specific Anti-CD19/CD3 BiTE^®^ Antibody for the Treatment of Acute Lymphoblastic Leukemia: Perspectives and Current Pediatric Applications

**DOI:** 10.3389/fonc.2014.00063

**Published:** 2014-03-31

**Authors:** Lindsey M. Hoffman, Lia Gore

**Affiliations:** ^1^The Center for Cancer and Blood Disorders, Children’s Hospital Colorado, School of Medicine, University of Colorado Cancer Center, Aurora, CO, USA

**Keywords:** immunotherapy, acute lymphoblastic leukemia, pediatric leukemia, drug development, bi-specific antibody, T-cell engager

## Abstract

Leukemia is the most common childhood malignancy and acute lymphoblastic leukemia (ALL) represents the largest sub-type. Despite remarkable improvements over the last 40 years, standard therapy fails in 10–20% of newly diagnosed patients. Survival for children with relapsed ALL is poor, and the development and implementation of novel therapeutic strategies in pediatric ALL are critical to further advancements. Immunotherapeutic approaches have been central to more novel ALL therapies. However, more recent innovation in antibody engineering has improved potency and efficacy, and antibody–drug conjugates (ADCs) are an especially attractive option in severely immunocompromised patients. An even more sophisticated antibody design is that of bi-specific T-cell engaging or BiTE^®^ antibodies, which directly recruit effector T cells to augment the anti-neoplastic effect. This review focuses on blinatumomab, a bi-specific anti-CD19/CD3 antibody that has shown efficacy in adult patients with precursor B-ALL and is currently being evaluated in the pediatric setting.

## Introduction

Leukemia is the most common childhood malignancy, of which acute lymphoblastic leukemia (ALL) represents the largest sub-type, accounting for nearly 75% of newly diagnosed cases. Despite remarkable outcome improvement over the last 40 years, recent data suggest that standard therapy fails in 10–20% of newly diagnosed patients (1), and given its incidence, ALL remains the leading cause of cancer-related mortality under age 19 years (2). Survival for children with relapsed ALL is poor, reaching only 15% in those with early bone marrow relapse (3). While traditional salvage regimens, which involve intensive combination chemotherapy followed by allogeneic hematopoietic stem-cell transplant (HSCT), may cure a small proportion, inability to achieve sustained remission, and concern for cumulative treatment-related morbidity and mortality has prompted tremendous effort toward development and implementation of novel therapeutic strategies in pediatric ALL.

Increasing recognition of the heterogeneous mutational and epigenetic landscape of pediatric ALL has centered many novel therapeutic approaches on targeted molecular agents, as demonstrated in Philadelphia-positive (Ph+) ALL. The prototype of modern therapeutic advancement in this realm is the use of the BCR-ABL kinase inhibitor, imatinib, in Ph+ ALL, which has transformed today’s treatment approach and dramatically improved event-free survival in this leukemia sub-type (4). Despite this success, clinical translation of most novel therapeutic targets is slow, and development of resistance and up-regulation of escape pathways have proven formidable opponents to maintaining sustained response to such agents.

Immunotherapeutic approaches have more recently come to the forefront in ALL therapy. Given the reliability with which leukemia cells express surface antigens *not* expressed on normal tissue, monoclonal antibodies are being widely investigated. Unconjugated monoclonal antibodies, including rituximab (5, 6), alemtuzumab (7), and epratuzumab (8), have demonstrated utility in hematologic malignancies for several decades. However, more recent innovation in antibody engineering has improved potency and efficacy, including the creation of antibody–drug conjugates (ADCs), which link antibodies to various chemotherapeutics, radionucleotides, or toxins to enhance the cell killing with less reliance on active host immunity, an especially attractive option in severely immunocompromised patients. An even more sophisticated antibody design is that of bi-specific T-cell engaging or BiTE^®^ antibodies, which directly recruit effector T cells to augment the anti-neoplastic effect. Clinical use of bi-specific antibodies was first reported in 1995 (9); since that time, promising pre-clinical and early phase clinical work in a variety of human cancers has fortified interest in these constructs.

Key features of BiTE antibodies include the ability to redirect target cell lysis via T-cells at sub-picomolar concentration, the ability to activate T-cells to kill in the presence of target cells, and the ability to allow T-cells to lyse target cells in series (9). This chain of events leads to a triggered activation of T-cells that stimulates proliferation of CD4^+^ and CD8^+^ cytotoxic cells as long as target cells are available. Bi-specificity for CD3, for which stimulation is highly conditional, amplifies the T-cell signal (10, 11). Though T-cell auto-reactivity might be anticipated as potential complication of BiTE therapy, to date, no autoimmune disorders have been described in this context (10).

This review will focus on blinatumomab, a bi-specific anti-CD19/CD3 antibody that has shown efficacy in adult patients with precursor B-ALL (12) and is currently being evaluated in the pediatric setting.

## Blinatumomab Pharmacology

Blinatumomab is a 55 kDa-fusion protein comprised of two single-chain antibodies to CD19 and CD3, recombinantly joined by a flexible, non-glycosylated five-amino acid non-immunogenic linker that affords a very short distance between arms (10, 13, 14). CD19 is an attractive target in malignancies of B-cell origin, given its stable, nearly ubiquitous expression in B-cell malignancies (15). Furthermore, CD19 holds proposed importance in sustaining the malignant B-cell phenotype via mechanisms of proliferation, cell survival, and self-renewal (16–18). The mechanistic advantage of blinatumomab include its ability to draw malignant B-cells in close proximity to CD3-positive T-cells without regard to T-cell receptor (TCR) specificity or reliance on major histocompatibility complex (MHC) class I molecules on the surface of antigen presenting cells for activation. This non-specificity allows recruitment of a polyclonal T-cell population (14, 19) and circumvents a known mechanism of resistance to T-cell-based therapies through down-regulation of MHC class molecules (20). Co-binding of CD19 and CD3 leads to T-cell activation, marked by up-regulation of T-cell activation markers CD25, CD69, CD2, INFγ, TNFα, and IL-2, -6, and -10 (21). Cell lysis is mediated by secretion of perforin and various granzymes stored in the secretory vesicles of cytotoxic T-cells (22). *In vitro* data suggest that efficacy of blinatumomab is not compromised by low effector-to-target cell ratios (14, 19) or dependent upon T-cells, which may be limited in number in heavily pre-treated patients (23). Additionally, blinatumomab-activated T-cells appear to effectively induce serial target cell killing, as seen on video-assisted microscopy (14).

## Pharmacokinetics and Administration of Blinatumomab

Various schedules of blinatumomab administration have been investigated, including short intravenous (IV) infusion that was initially evaluated in a series of three adult phase I studies. Pharmacokinetic (PK) profiling in these studies at dose levels ranging from 0.75 to 13 μg/m^2^ revealed a short half-life of approximately 2 h and provided further insight into the occurrence of serial lysis, providing rationale for administration by continuous infusion (24). PK analysis of continuous infusion blinatumomab in adult patients with relapsed non-Hodgkin lymphoma (NHL) showed a linear dose curve and predictable drug levels throughout, making 24-h infusion the preferred schedule of blinatumomab administration. Though pediatric experience is limited, PK data from the first phase I study of blinatumomab in children indicate similar serum concentrations to those achieved in adults with attainment of steady state concentration within 48 h of continuous infusion (25, 26).

Given somewhat unique PK characteristics of blinatumomab, there are certain practical requirements of administration. In current clinical trials, blinatumomab is administered over a 28-day continuous infusion, followed by a 14-day rest period. Shorter rest periods between cycles have not been formally studied to date. Multiple cycles of administration have been safely delivered. Based on the side effect profile discussed below, hospital admission is currently required for close observation during treatment initiation, depending on the extent of disease burden and the stage at which patients are treated. Subsequently, with low disease burden and after the peak period of side effects has passed, patients may be discharged from hospital to continue treatment in the outpatient setting.

## Safety and Tolerability of Blinatumomab

Extensive pre-clinical characterization of blinatumomab, including *in vivo* work in both mice (27) and chimpanzees (28) led to first-in-human studies in 2001. A series of three adult phase I studies evaluated blinatumomab given as a short IV infusion at dose levels from 0.75 to 13 μg/m^2^ in 21 patients with relapsed/refractory NHL and one patient with chronic lymphocytic leukemia (CLL). The most commonly observed adverse events (AEs) were pyrexia, rigors, and fatigue, of which most were mild and all were reversible (24). More clinically pertinent AEs involved CNS toxicities (including headache, aphasia, ataxia, somnolence, tremor, disorientation, seizure), cytokine release syndrome (CRS), and leukopenia- and neutropenia-related infection, which in the absence of objective responses, prompted early termination of these initial studies. A subsequent phase I dose-escalation study of continuous administration blinatumomab showed similar AEs of pyrexia, chills, and leukopenia. However, at dose levels up to 60 μg/m^2^/day, CNS toxicity was less severe and CRS was not observed, further supporting continuous infusion as the preferred method of administration (29). After a dose finding run-in portion, larger phase II studies in adult patients with minimal residual disease positive (MRD^+^) ALL utilized a dose of 15 μg/m^2^/day and demonstrated toxicity profiles comparable to the ones in NHL but with lower incidence of CNS toxicity in patients with MRD^+^ ALL after first line induction and consolidation (12). In patients with relapsed/refractory ALL, the incidence of CNS toxicity and CRS was higher than in patients with MRD^+^ ALL, in whom no clinical CRS was observed (12, 30). Several current trials in adult patients are exploring flat dosing of blinatumomab at 28 μg/day. In relapsed/refractory ALL, a pre-phase using a lower dose of blinatumomab is employed in an attempt to prevent or ameliorate CRS prior to dose-escalation.

Preliminary data from the first and only pediatric phase I study to date suggest substantial similarities among the pharmacological, toxicity, and response profiles described in adults. This study, which evaluates dose levels ranging from 5 to 30 μg/m^2^/day in children and adolescents with relapsed/refractory ALL, determined a maximum tolerated dose (MTD) of 15 μg/m^2^/day and a recommended dose of 5 μg/m^2^/day in the first week, followed by escalation to 15 μg/m^2^/day beginning on day 8 and for all subsequent days of infusion (25, 26). There were two fatal dose-limiting toxicities (DLTs) noted at higher dose levels (one CRS, one respiratory failure). Similar to adult studies, greater than 80% of patients at all dose levels, including 86% of patients at the MTD (*n* = 7), experienced AEs of grade 3 or higher. The most common AEs at the MTD were pyrexia (100%), hypertension (71%), and headache (57%). Nervous system disorders, typically mild, were common, occurring in 57% of patients at the MTD. However, all five patients (100%) treated at 5 μg/m^2^/day experienced some degree of reversible neurologic toxicity, suggesting a non-linear relationship. CRS was observed in 7 of 23 patients (30%) in this study and was more common at higher dose levels and during the first cycle of therapy, which likely correlates to larger disease burden, as reported in adults (30). In light of the incidence and timing of CRS, an altered blinatumomab dosing schedule of 5 μg/m^2^/day in the first week and 15 μg/m^2^/day in the subsequent 3 weeks of the first cycle was evaluated and determined to be safe and tolerable in an expanded pediatric cohort of patients with ≥25% bone marrow blasts. This dose is being evaluated in a phase II cohort currently.

Though the majority of patients treated with blinatumomab develop mild inflammatory symptoms related to T-cell activation at initiation of therapy, CRS is a more severe condition characterized by “flu-like” symptoms, such as fever, chills, and headache, and the potential for hemodynamic instability, bleeding, capillary leak syndrome, and respiratory compromise (31, 32). While symptom severity varies, grade 3 or higher CRS has been observed in a small percentage of adult patients (32). Release of inflammatory cytokines IL-2, IL-6, IL-10, INFγ, and TNFα has been demonstrated in adult and pediatric patients (31, 33). Data from the recently completed phase I portion of the first pediatric study correlated early elevation and subsequent decline of inflammatory cytokines with clinical symptoms of CRS, most particularly IL-6 and IL-10, and to a lesser degree INFγ (25, 26). The most recent adult ALL study failed to associate degree of elevation of inflammatory cytokines to target B-cell frequency in the blood or bone marrow (33) or to patient outcome (12). Acquisition and maturation of additional pediatric data are necessary to further evaluate this association in children.

To dampen the pro-inflammatory response associated with blinatumomab therapy, current clinical trials mandate co-administration of dexamethasone at the initiation of therapy, which has been shown in cell culture experiments to effectively reduce cytokine concentration without affecting T-cell activation or the cytotoxic potential of blinatumomab against malignant B-cells (21). An additional therapeutic option to negate more severe symptoms of CRS is the IL-6 receptor antibody tocilizumab, which recently showed clinical benefit in a pediatric patient with life-threatening CRS (31). This addition to the therapeutic arsenal offers an additional option for symptom control in patients with very severe CRS that is not sufficiently managed with steroids.

## Clinical Efficacy of Blinatumomab

Efficacy of blinatumomab in B-cell driven malignancies was first demonstrated in adult relapsed NHL. A dose-escalation phase I study by Bargou et al. in 2008, reported “major response” in 11 of 38 patients, including 4 and 7 patients with complete and partial responses, respectively (29). This study, which evaluated dose levels from 0.5 to 90 μg/m^2^/day, determined an MTD of 60 μg/m^2^/day in adults. However, in those with bone marrow involvement, doses as low as 15 μg/m^2^/day were effective, providing dosing rationale for the first study of blinatumomab in MRD^+^ precursor B-cell ALL.

Minimal residual disease has emerged as perhaps the most important prognostic factor in the majority of both pediatric (34, 35) and adult (36) ALL cases. A phase II study by Topp et al. evaluated the efficacy of blinatumomab at 15 μg/m^2^/day in 21 patients with MRD positive ALL (12). Of 20 evaluable patients, 16 (80%) responded with negative MRD at the end of the first cycle, including 12 patients who had persistent MRD after previous induction and consolidation. Follow-up of these patients showed 61% relapse-free survival at a median of 33 months (37).

A subsequent study evaluated response to blinatumomab in relapsed/refractory precursor B-cell ALL (30). Though the initial starting dose was 15 μg/m^2^/day, as in the prior study, the dose finding run-in part demonstrated a modified two-level dose of 5 and 15 μg/m^2^/day for the first week and subsequent 3 weeks, respectively, as the optimal dose based on increased toxicity (particularly CRS) in patients who received 15 μg/m^2^/day throughout the cycle. This dose was used for the Simon two-Stage part of the trial. Complete response was observed in 25 of 36 patients (69%), and of those, 22 of the 25 responders (88%) achieved MRD negative status (<10^−4^ cells by PCR). Response was higher amongst those in first versus second or greater relapse.

In 2010, Handgretinger et al. described the first pediatric experience with blinatumomab in three children with multiply relapsed precursor B-cell ALL following allogeneic HSCT (38). Complete morphologic response with negative MRD was observed in all patients after the first cycle of therapy, which consisted of 24 h continuous infusion blinatumomab at 15 μg/m^2^/day for 4–6 weeks. These responses in addition to compelling adult data prompted initiation of the first phase I/II study of blinatumomab in children, currently underway in Europe and North America through the International Berlin–Frankfurt–Munster (iBFM) study group and the Children’s Oncology Group (COG), respectively, which is described above. Though limited within the context of a phase I trial, published objective responses were noted in 43% of patients, including partial response in 1 patient (4%) and complete morphologic response with negative MRD (<10^−4^ cells detectable by PCR) in 39% of patients. The phase II portion of this study to more completely evaluate the efficacy and safety of this regimen in children is on-going.

## Commentary and Conclusion

The biologic complexity of pediatric ALL necessitates multifaceted therapeutic approaches. While the backbone of ALL therapy remains cytotoxic chemotherapy with an ever-increasing role for targeted molecular agents, blinatumomab is a new addition to the arsenal against adult and pediatric ALL, in which integration of immunotherapy stands to further improve outcomes in both the upfront and relapsed settings. As experience with blinatumomab in the setting of refractory and relapsed pediatric ALL continues to grow, so does the level of comfort of managing the unique side effect profile of this agent. What has yet to be defined is whether or not there is a way to predict which patients will respond *a priori*, whether or not abrogation of the cytokine release or pre-treatment with steroids diminishes the efficacy in children, and whether or not the degree of pre-treatment or specific or cumulative prior therapies affects the potential for response. To date, some patients who have progressed on or following blinatumomab administration have gone on to chimeric antigen receptor (CAR) T-cell-based therapies or other clinical trials with investigational agents. At least one patient who did not respond to blinatumomab was able to achieve remission with CAR-T cell administration (Personal Communication, S. Grupp), although this has not been widely or formally reported in the pediatric population to date. The optimal placement of immunotherapy with blinatumomab and/or other conventional and immune modulating approaches has yet to be determined. Similarly, integration of blinatumomab into combination regimens will need to account for the unique toxicity profile to ensure optimal safety for patients, as well as the best efficacy potential.

Further clinical investigations to begin to answer some of these questions and determine the optimal patient population, sequence in therapy, and potential combination treatments will better elucidate the possible role of blinatumomab in future treatment of childhood ALL.

## Conflict of Interest Statement

Lia Gore is the principal investigator of a clinical trial using blinatumomab in pediatric patients. She is a member of Amgen’s Scientific Advisory Board for blinatumomab.
